# BANK1 and BLK Act through Phospholipase C Gamma 2 in B-Cell Signaling

**DOI:** 10.1371/journal.pone.0059842

**Published:** 2013-03-26

**Authors:** Manuel Bernal-Quirós, Ying-Yu Wu, Marta E. Alarcón-Riquelme, Casimiro Castillejo-López

**Affiliations:** 1 Centro de Genómica e Investigación Oncológica, Pfizer-Universidad de Granada-Junta de Andalucía, Granada, Spain; 2 Arthritis and Clinical Immunology Program, Oklahoma Medical Research Foundation, Oklahoma City, Oklahoma, United States of America; Hungarian Academy of Sciences, Hungary

## Abstract

The B cell adaptor protein with ankyrin repeats (BANK1) and the B lymphoid tyrosine kinase (BLK) have been genetically associated with autoimmunity. The proteins of these genes interact physically and work in concert during B-cell signaling. Little is know about their interactions with other B-cell signaling molecules or their role in the process. Using yeast two hybrid (Y2H) we sought for factors that interact with BANK1. We found that the molecular switch PLCg2 interacts with BANK1 and that the interaction is promoted by B-cell receptor (BCR) stimulation. We found further that the kinase activity of BLK enhanced BANK1- PLCg2 binding and that the interaction was suppressed upon BLK depletion. Immunoprecipitation and mutational analysis demonstrated that the interaction between BANK1 and PLCg2 was dependent on specific tyrosine and proline residues on the adaptor protein. Our results provide new information important to understand the role of these two genes in basic B-cell physiology and immune-related diseases.

**Table 1:** Clones isolated in the yeast two-hybrid screen using the full-length BANK1.

Description	Frame	AA	%	Clones
APG4 autophagy 4 homolog B	IF	323	82	
Baculoviral IAP repeat-containing 6	F1	291	6	
CD163	IF	330	29	
Filamin A	F1	157	6	
FYN kinase related to src	F2	118	22	
Lipin 1	IF	124	14	
Purinergic receptor P2Y, G-protein coupled, 8	F1	175	48	
Phospholipase C, gamma 2	IF	339	27	A-20
Phospholipase C, gamma 2	IF	372	29	A-34

Clones isolated in the yeast two-hybrid screen using the full-length BANK1 (amino acids 1–785) as bait. The frame indicates if the coding sequences are in the same frame as Gal4-Activating Domain. In general polypeptides not having an in-frame (IF) position are not considered of biological interest. However, some of the proteins expressed from F1 or F2 can be translated in the correct frame, due to the existence of natural frame-shift events during translation in yeast. AA indicates the length of the clone in number of amino acids and (%) the percentage of frame corresponding to the annotation in GenBank.

## Introduction

Traditionally, molecules that regulate B-cell signaling have been classified either as activators or inhibitors of B cell activation. However experimental data has shown a more complex level of functional interaction. For example it has generally been assumed that kinases acting in the initiation of B-cell signaling such the Src-family tyrosine kinases are predominantly activating factors [1]. But, their ability to negatively regulate the signaling pathway through phosphorylation of inhibitory molecules is exemplified by the B-cell hyperactivation shown in the Src- kinase *lyn* deficient mouse (*lyn*-/-) [2], [3]. On the other hand, over expression of genes encoding for inhibitory cell-surface molecules such the phosphatase CD45 enhanced B cell activation leading to autoimmunity [4]. In this case, the hyperactivation was the result of the phosphatase activity on the negative regulatory C-terminal tyrosine found in the Src-family kinases [5], [6]. The B-cell adaptor protein with ankyrin repeats (BANK1) has been consistently associated with the autoimmune diseases such as SLE and systemic sclerosis [7], [8] BANK1 acts in the B-cell signaling pathway but lacks enzymatic activity. It does contain a number of sites of tyrosine phosphorylation and proline-rich motifs that could contribute to the interaction to proteins harboring SH2 and SH3 domains, respectively. BANK1 possesses two ankyrin repeats and a conserved region denominated Dof-BCAP-BANK motif involved in dimerization [9], [10]. BANK1 is extensively phosphorylated upon B-cell antigen receptor (BCR) engagement. In the chicken DT40 cell line this phosphorylation is dependent on the expression of the spleen tyrosine kinase (SYK) and independent of the expression of the kinases of the Src family LYN or BTK. Although the phosphorylation of BANK1 does not require LYN, the phosphorylation of BANK1 enhances binding to LYN and promotes tyrosine phosphorylation of IP3R2 leading to release of calcium from intracellular stores [11]. The analysis of the BANK1 knock-out mouse indicates that the protein acts as a negative regulator of CD40-mediated Akt activation. The animals show slight increase of germinal center formation and overproduction of IgM antibodies to T-dependent antigens [12]. Although the involvement of BANK1 in the BCR and CD40-mediated pathways is consistent with a function for BANK1 as a canonical adaptor molecule, the phenotypes observed in over-expressing or knock-out cells is not understood.

Phosphoinositide-specific C phospholipases (PLC) are one of the major group of cell-signaling switch molecules due to their role in the formation of the second messenger inositol 1,4,5-trisphospate (IP3) and diacylglycerol (DAG). There are six families of PLC enzymes that differ in their amino acid sequence and structural organization [13]. The two members of the branch corresponding to the PLCg family have inserted within the catalytic core, two Src homology 2 (SH2) domains and one SH3 domain. The C-terminal SH2 domain is a critical determinant for auto-inhibition [14], while translocation to lipid membranes is required for full enzymatic activity [15]. PLCg1 is ubiquitously expressed while PLCg2 is most highly expressed in cells of hematopoietic origin and play a critical role in the regulation of the immune system [16], [17]. A gain-of-function mutation in murine PLCg2 increased its membrane stability and lead to severe autoimmunity [18]. Recently, a human dominantly inherited phenotype linked to deletions of the autoinhibitory domain of PLCg2 was reported. The deletions lead to constitutive phospholipase activity and autoimmune disease [19]. Thus, mutations of PLCg2 that affect its activity or its temporal location or by extension, variants in associated molecules could lead to complex immunological phenotypes with very different manifestations, such as inflammation, cold urticaria or signs of autoimmunity such as autoantibody formation.

**Figure 1:**
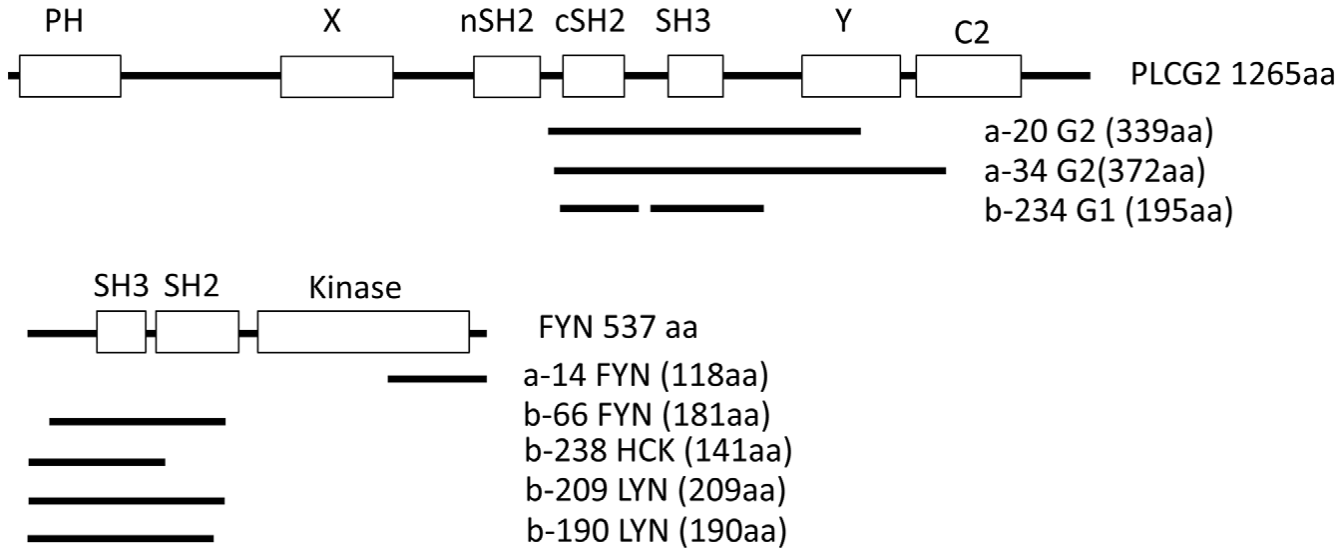
Clones belonging to the phosphoinositide-specific phospholipase C and the family of Src kinases isolated in two yeast two-hybrid screens. (A) Representation of PLCg2 modular structure and the coding region of the clones identified in the Y2H. Clones named a- correspond to the first screen using as bait the full-length BANK1, clones starting with b- are the ones identified in the screen with the non autoactivating truncated protein BANK1 (331–785). The clone b-234 has a deletion of 25 aa between the cSH2 and SH3 domains. (B) Structure of the Src kinase FYN and the isolated clones belonging to this family of non- receptor kinase. PH: Pleckstrin homology domain, involved in the recruitment to membranes by binding to phosphatidylinositol containing lipids. X and Y are the two halves of the catalytic isomerase. SH2 (Src homology 2) conserved domain that typically binds to phosphorylated tyrosine residues. SH3 (Src homology 3) usually binds to proline-rich motifs. The C2 motif is present in many proteins that interact with membranes and are frequently involved in calcium dependent phospholipid binding and membrane targeting processes.

Recently we identified that BLK a kinase of the Src family was an interacting partner of BANK1[20]. BLK belongs to the Src-family of tyrosine kinases that include also the related proteins SRC, LYN, FYN, YES, HCK, FGR and LCK [21]. All share a modular structure composed of a N–terminal with attachment sites for fatty acid modifications, a unique region, a Src-homology 3 (SH3) domain, a Src-homology 2 (SH2) domain, a tyrosine kinase domain and a C-terminal kinase inhibitory domain (Figure 1). Src kinases bind to the cytoplasmic portion of surface receptors and participate in diverse signaling pathways. Although their catalytic activity is apparently redundant, each member might achieve a unique function due to tissue-specific expression and sub-cellular localization, which is mainly determined by the fatty acid modifications.

In this study we have identified and characterized the interaction of the adaptor protein BANK1 with the molecular switch PLCg2 and suggest a role for the kinase BLK in this process. We show that the interaction between PLCg2 and BANK1 is promoted by the engagement to the BCR and through the binding to proline rich-motifs and phosphorylation of tyrosine residues on the BANK1 adaptor molecule. The formation of the BANK1-PLCg2 complex is modulated by the sub-cellular location and the kinase activity of BLK. Our data suggest that BANK1 and BLK function to link BCR-mediated signaling to the formation of intracellular second messengers through PLCg2.

## Results

### Identification of protein partners interacting with the B-cell scaffold protein with ankyrin repeats (BANK1)

Two independent yeast two-hybrid screens were carried out to identify interacting partners of BANK1. In the first one, we used as bait the full-length form of BANK1 (aa 1–785). Due to the high autoactivity of the full-length BANK1 construct, we performed a mapping of BANK1 domains to identify fragments that do not or mildly autoactivate the transcription of the reporter gene in the yeast two-hybrid system (Figure S1). Once the non-activating domains were identified, we performed an additional screen with the N-terminal truncated form of BANK1 (aa 331–785).

The screen with the full-length form of BANK1 identified 9 clones with good or moderate confidence in the interaction (Table 1). The interaction found with the Src kinase FYN rendered the results of the screen reliable. The hypothesis preceding the screen was to recover prey clones coding for conserved Src family of kinases because it has been previously shown that BANK1 interacts physically *in vivo* with two related Src kinases, namely LYN [11] and BLK [20]. The higher confidence for interaction was however obtained with the phospholipase C-gamma 2 (PLCg2). Two independent clones coding for the regulatory region specific for the PLCg family were recovered. Both clones code for the carboxy terminal SH2 domain (cSH2), the complete SH3 domain and one of clones included the carboxy terminal catalytic Y-core (Fig.1)

The second screen with the truncated form of BANK1 (aa 331–785) produced high confidence interactions, which suggests that this fragment of BANK1 is, at least partially well folded. The complete set of 95 prey proteins is listed in Table S1. The higher scores in this screen were given to the genes G22P1 coding for the Ku70 protein, [22], [23] and the genes PSAP and Saposin C coding for the saposin precursor and the mature Saposin C form, respectively [24]. In this screen, we identified once again fragments as prey clones coding for the SH2 and SH3 domains of the related Src kinases LYN, FYN and HCK (Figure 1). In addition a single clone coding for a polypeptide from PLCg1 was found. The aa sequence is highly homologous to PLCg2 and correspond to aa 647–843 that comprise the cSH2 domain and the complete SH3 domain. Surprisingly, the clone had a 25 aa deletion that removes two tyrosine residues previously implicated in phosphorylation-dependent activation of the lipase [14], suggesting that this domain is dispensable for the binding to BANK1. With the only exception of the clone A-14 coding the kinase domain of FYN (Figure 1), all the recovered clones belonging to PLCg and Src-kinase families expressed the SH3 and a truncated SH2 domain, which indicates that these motifs are implicated in the interaction with BANK1.

### Ectopically expressed BANK1 co-localizes with Phospholipase C-gamma 2 (PLCg2)

To confirm the interaction between BANK1 and the recovered Y2H clones we expressed ectopically the proteins and performed co-localization studies. In addition to PLCg2, two other prey genes were chosen for validation: The scavenger receptor CD163 and the autophagy related protease ATG4b, also called ATG4B (Table 1) [25], [26], [27]. ATG4b was chosen because the length of the clone and the frame of the lecture were optimal.

**Figure 2:**
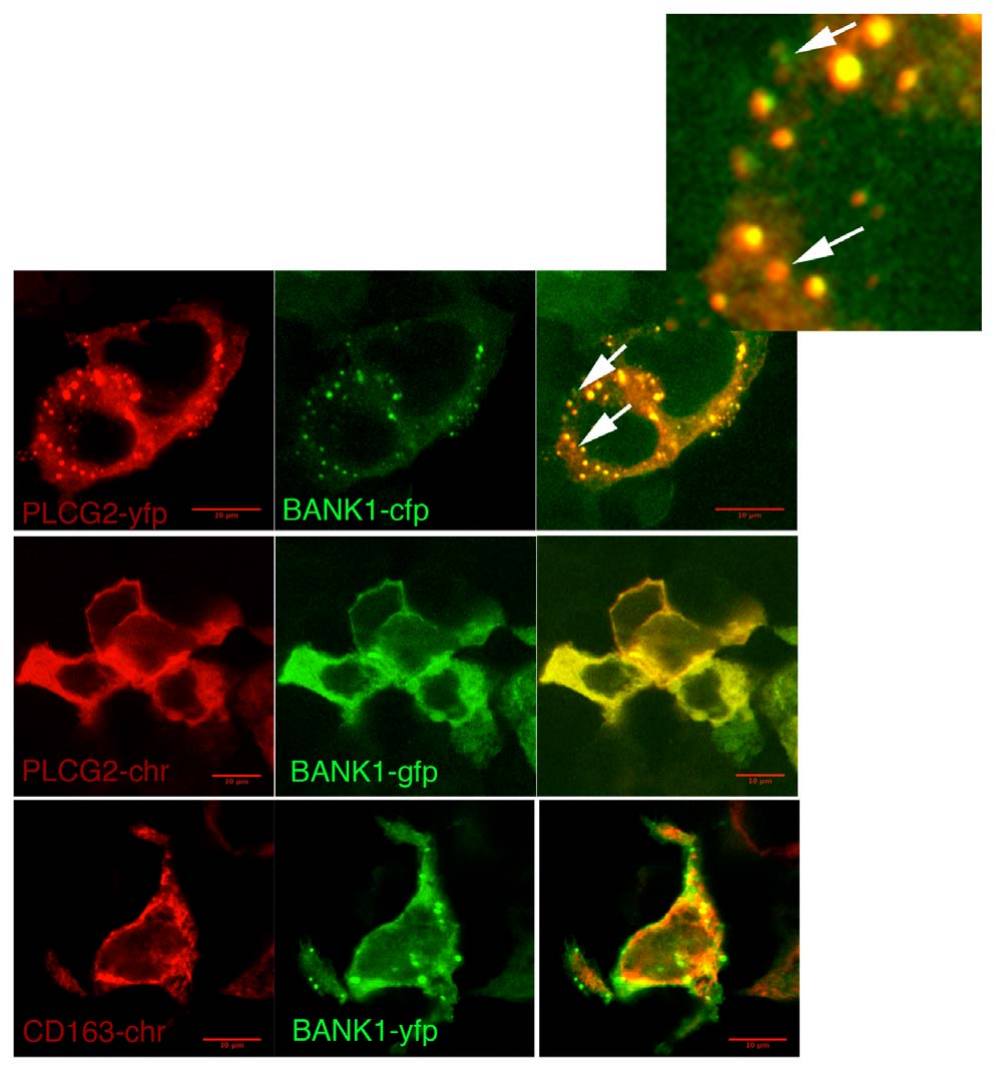
BANK1 and PLCG2 co-localize in cytoplasmic compartments. Confocal images of HEK293 cells co-expressing fluorescently tagged proteins. BANK1 is a cytoplasmic adaptor protein that shows a punctate and homogeneous pattern of distribution. PLCg2 shares the same sub-cellular compartments when co-expressed with BANK1. Upper row, co-expression of PLCg2 and BANK1 showing sub-cellular co-localization in punctate structures. Although a perfect co-localization between the two proteins is observed, there are dots (indicated by arrows, both in the merge image and in the amplification at the right upper corner) in which the ratio of the two proteins is reversed. Cross-talks between light channels were further ruled out by assessing the emission on cells expressing only one fluoresce protein and excitated sequencially with both lasers. Middle row, images showing co-localization between PLCg2 and BANK1 in a homogeneous cytoplasmic distribution. Lower row, images showing only partial co-localization between the cytoplasmic scavenger receptor, CD163 and BANK1. Scale bar: 10 µm.(-yfp; yellow fluorescence protein. –cfp; cyan fluorescence protein. –chr; cherry fluorescent protein. –gfp; green fluorescent protein).

Co-expression of BANK1 and PLCg2 showed perfect co-localization while CD163 and ATG4b show only partial co-localization with BANK1 (Figure 2 and Figure S2). BANK1 is a cytoplasmic protein that when ectopically expressed shows a variable pattern of expression. BANK1 distributes homogeneously through the cytoplasm and under certain circumstances concentrates in punctate structures [28]. Cells showing an evenly distributed cytoplasmic pattern of BANK1 do present an equally distributed PLCg2. Likewise, in cells where BANK1 showed punctate structures, PLCg2 co-localized with the majority of the dots. The inset in Figure 2 shows that individual dots have uneven quantity of each protein which is important technically because it indicates that we have unmixed detection channels and render our results highly reliable.

The expression of CD163 shows a cytoplasmic distribution with a reticulate and punctate pattern that partially co-localizes with BANK1 (Figure 2). In our experiments, the sub-cellular location of CD163 is identical to the distribution of the endogenous CD163 protein [29], suggesting the expression system and the addition of the fluorescent tag did not interfere in the localization of the exogenously expressed proteins. At this point, we concluded that there were no obvious sub-cellular constrains for the interaction between BANK1 and PLCg2 and both proteins shared cellular compartments.

### BANK1-PLCG2 complex formation is transient and induced by IgM stimulation

**Figure 3:**
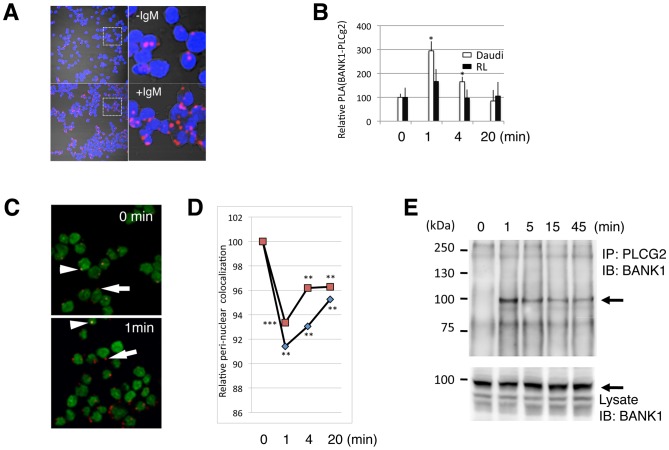
BANK1-PLCG2 complex formation is transient and induced by IgM stimulation. (A) Confocal images of Daudi B cells showing increase molecular proximity between endogenous BANK1 and PLCg2 proteins upon anti-IgM stimulation. The staining was done using *in situ* a PLA protocol with rabbit anti-BANK1 (ET-52) and mouse anti PLCg2 (Abcam). Nuclei were stained with DAPI in blue. The confocal images (PLA signals) were taken with a pinhole of 2.5 (Zeiss Plan-Apochromat 63× oil objective). Upper panel, non-stimulated cells. Low panel, cells stimulated for 1 minute with anti-human IgM-F(ab')2. To the right are shown digital magnifications. (B) Time variation of PLA BANK1-PLCg2 interaction upon stimulation with anti-IgM in two human B-cell lines, the Burkitt´s derived Daudi and the non-Hodgkin´s lymphoma derived RL. Results are shown as the mean from three independent experiments. Error bars represent the SD from the mean. *P<0,05 based on Student´s test comparing stimulated cells versus time 0 (non stimulated cells).(C) Merge confocal images (pinhole = 1) of Daudi cells showing the nuclei in green and the BANK1-PLCg2 PLA interaction in red to permit co-location analysis. PLA signals close to the nucleus appeared as yellow dots (arrowheads) and when localize in the periphery of the cell as red dots (arrows). (D) Time course upon anti-IgM stimulation of co-localization between the PLA signal and the nucleus. Quantification was done using the overlap coefficient after Manders because this coefficient is insensitive to differences in signal intensities between channels. After one minute of stimulation the co-localization decreases suggesting a translocation of the BANK1-PLCg2 complex. The graft shows two independent experiments, each point represents the analysis of at least 300 cells.**,P<0,01;***P<0,001 based on Student´s test comparing stimulated cells versus time 0 (non stimulated). (E) Immunoprecipitation of the BANK1-PLCg2 complex in Daudi cells. Anti-PLCg2 immunoprecipitates (above) and total cell lysate (below) were analyzed by immunoblotting with anti-BANK1 antibody (BANK1-ET-52). The position of the BANK1 protein is indicated by arrows.

In order to validate the physical interaction between BANK1 and PLCg2 proteins, we performed co-immunoprecipitation with the ectopically expressed fusion proteins. The co-immunoprecipitation between BANK1-PLCg2 was low and some times failed when we used HEK293 cells expressing both proteins. We reasoned that protein interactions could be weak or dependent on posttranslational modifications such as phosphorylation that eventually, might not occur in HEK293 cells because of the lack of the appropriate kinases and the need of BcR-mediated signaling. We tested the first alternative using an *in situ* Proximity Ligation Assay (PLA) that allows measuring weakly connected proteins. The technique detects interacting proteins using a pair of antibodies raised in different species linked to complementary DNA oligonucleotides. Close proximity of the antibodies allows ligation and subsequent amplification of the DNA probe. Single interacting protein events are visible as bright dots when viewed with a fluorescence microscope [30]. Optimization of the PLA assay was done using exogenous expression of BANK1 and PLCg2 in HEK293 cells and also using the endogenous molecules in EBV immortalized B-cells (Figure S4). Next, we determined the kinetic of BANK1-PLCg2 PLA interaction in Daudi and follicular lymphoma derived RL B-cell lines upon IgM stimulation. In non-stimulated cells (Figure 3A, upper row) we could detect only few interactions but still above the noise level obtained in the negative control HEK cells. After stimulation with anti-IgM the interaction of the two proteins increased (Figure 3A-lower row) indicating that both proteins translocated to close proximity in response to anti-IgM stimulation. Both cells lines showed similar kinetics, increasing the interaction at one minute after stimulation and returning to initial levels after twenty minutes. The variation in PLA signal was stronger in Daudi cells (P-value = 0.019) compared with RL cells (P-value = 0.16) based on Student´s t test comparing stimulated cells versus time 0 (Figure 3B).

Because full enzymatic activity of PLCg2 requires proximity to the plasma membrane [15], we tested whether the stimulation with IgM leads to the translocation of the BANK1-PLCG2 complex away from their perinuclear location in Daudi cells. Using co-localization coefficients between the PLA signal and the DAPI staining we quantified the localization of the BANK1-PLCG2 interactions. In non-stimulated cells (0 min), the PLA signals are mostly localized close to the nucleus, resulting in a merged image with yellow spots, shown by arrows (Figure 3C). After one minute of IgM stimulation the PLA signals lose their perinuclear location appearing as red spots in the merge image as shown by the arrowheads (Figure 3C below). The translocation of the PLA signal was quantified in two independent experiments (Figure 3D). In non-stimulated cells, the few BANK1-PLCG2 interactions are close to the nuclei, they lose the perinuclear localization at one minute of stimulation and at 20 minutes return to a position close to the nuclei. PLCg2 is required for BCR-induced spreading and formation immunological synapses [31], our current experimental settings are unable to discriminate whether the translocation of the BANK1-PLCg2 signals is due to the translocation to the plasma membrane or a consequence of the cellular spreading in response to BCR engagement.

The dynamics of the interactions upon BCR cross-linking was further addressed using conventional immunoprecipitation methods. Figure 3E shows that in resting B-cells the interaction between BANK1 and PLCg2 is negligible while an evident immunoprecipitate was obtained upon stimulation. The reverse immunoprecipitation using BANK1 antibody produced similar results (Figure S5). Based on these data we concluded that BANK1-PLCg2 interaction is transient and inducible upon BCR stimulation.

### The kinase activity and the lipidation of BLK contribute to the BANK1-PLCg2 interaction

**Figure 4:**
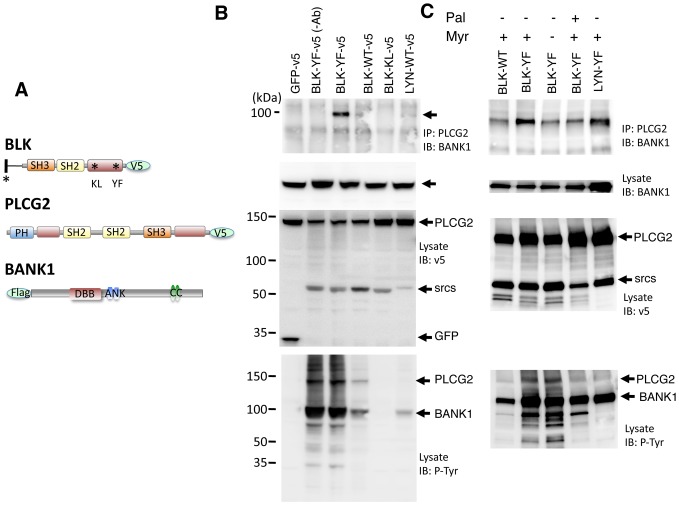
The constitutive-active form of BLK enhances the binding between BANK1 and PLCG2. (A) Schematic representation of the constructs used to study the association between BANK1 and PLCg2 in transfected HEK293 cells. The constructs coding for wild-type forms of BLK, LYN, GFP and PLCg2 or the indicated mutated forms were fused to the epitope V5 at the C-termini. BANK1 was targeted with the Flag epitope at the N- terminus. The catalytic domains of BLK and PLCg2 are shown in red. The kinase dead form (BLK-KL-v5) has a substitution K (lysine) to L (leucine) at position 269 and the constitutively active form (BLK-YF-v5) has a Y501F substitution that prevents the phosphorylation of the inhibitory tyrosine. The lipidation in the amino terminal of BLK is indicated as back line. The myristoylation site was deleted by G2V substitution (glycine to valine) and the addition of an extra by palmitoylated site by L3C substitution (leucine to cysteine). The Src homology 3 domains (SH3) that bind to proline-rich motifs are drawn in orange and the SH2 domains in yellow. The Pleckstrin homology domain (PH) that binds to phosphatidylinositol lipids is shown in blue. In BANK1 are shown the Dof/BCAP/BANK (DBB) motif (amino acids 199–327), the double ankyrin repeat-like (ANK) motifs (amino acids 339-402) and the putative coiled coil (CC) region (amino acids 677–705). (B) HEK293 cells were transiently co-transfected with plasmids coding for the wild-type form of BLK, its functionally mutated forms (KL and YF), LYN or GFP in addition to plasmids expressing BANK1 and PLCg2. The lysates were immunoprecipitated using anti-PLCg2 antibody (above) and immunoblotted sequencially with anti-BANK1 antibody, anti-V5 to detect PLCg2, Srcs kinases and GFP and anti-phosphotyrosine antibody. (C). Mutation of lipidation sites of the kinases influence the formation of the BANK1-PLCg2 complex and the overall tyrosine phosphorylation on PLCg2. The blots were interrogated as in B.

It has been shown that BANK1 is extensively tyrosine phosphorylated upon BCR stimulation [11]. It was thus very likely that the interaction between BANK1 and PLCG2 required phosphorylation of the adaptor protein. So far two kinases have been described as partners of BANK1, the Src kinase LYN originally used to isolate BANK1, and BLK, a similar Src kinase-specific of B cells that binds to BANK1 upon stimulation through the BCR [20]. To test directly that tyrosine phosphorylation of the adaptor protein enhances the BANK1-PLCG2 interaction, we co-expressed the interacting proteins, BANK1 and PLCg2 with the BLK and LYN kinases as well as with mutant forms of BLK (Figure 4). Complex formation was observed when BANK1 and PLCG2 were co-expressed with the constitutive active form of BLK (YF), Figure 4B, lane 3. Co-expression with the wild type (WT) kinases generated a weak but visible precipitate, lanes 4 and 6. The immunoprecipitate was absent when co-transfection was done with the kinase dead form of BLK (lane 5) or when using a protein lacking kinase activity (GFP), lane 1. Quantification in various independent experiments showed stronger immunoprecipitates using BLK-WT versus Lyn-WT, suggesting that BLK could be more specific than Lyn in the BANK1-PLCg2 interaction. However, because the expression of Lyn constructs was consistently lower we were unable to address adequately the contribution of each kinase to the BANK1-PLCg2 interaction; see for example the lysate, IB-v5 (Figure 4B). We observed nevertheless a difference in tyrosine phosphorylation of PLCg2; see lysate, IB-P-Tyr (Figure 4B). While the expression of BLK-WT phosphorylates PLCg2, the LYN-WT does not (compare lanes 4 and 6 in Figure 4B, IB: P-Tyr). To address if the sub-cellular localization of the kinases could influence the BANK1-PLCG2 interaction we constructed expression vectors with mutated lipidation sites (Figure 4C). Palmitoylation and myristoylation at the amino terminal residues of the Src-family tyrosine kinases is a major determinant for their intracellular distribution and trafficking [32]. BLK is myristoylated at the residue G2 and Lyn is myristoylated at G2 and palmitoylated at C3. The BANK1-PLCg2 interaction decreased when the constitutive active form of BLK lacked the myristoylation site or when an additional palmitoylation site is added. The last mutation mimics the lipidation pattern of Lyn, which suggests that single myristoylation of the kinase favors the BANK1-PLCg2 interaction (compare lanes 2, 3 and 4 of top panel of Figure 4C). Accordingly with this result, using the Lyn construct harboring the lipidation pattern of BLK renders a large amount of precipitate (lane 5 of top panel of Figure 4C), Thus both the kinase activity of BLK and its proper lipidation contributed to the specificity of BLK in the BANK1-PLCg2 interaction.

### Silencing of BLK reduces the association between BANK1 and PLCg2

**Figure 5:**
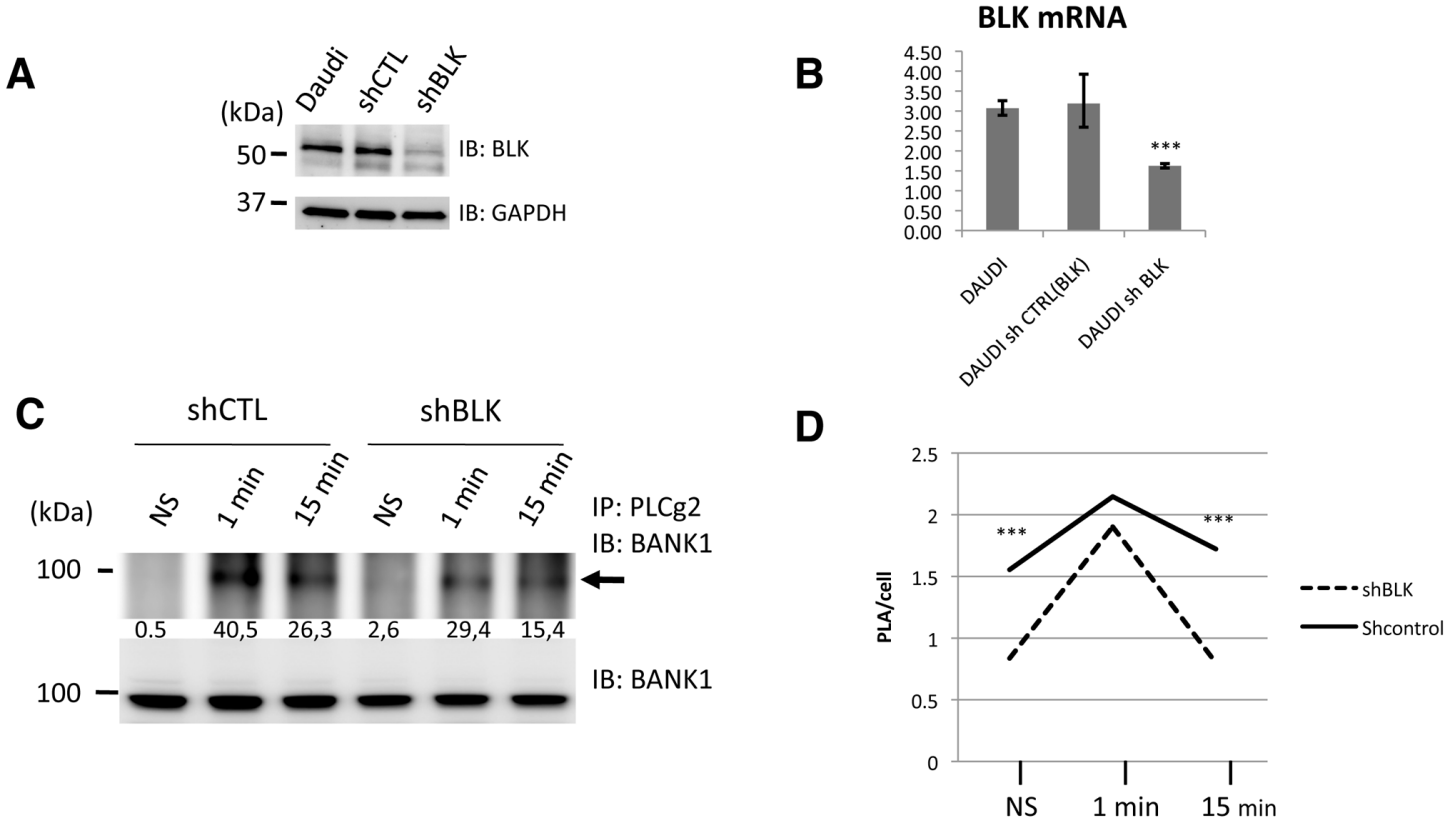
Silencing of the BLK kinase leads to alteration of the association between BANK1 and PLCg2. (A) Immunoblot of extracts derived from human Daudi B-cells showing efficient silencing of endogenous BLK protein. The cell line shBLK was transduced with lentivirus coding for small hairpin RNAs targeting the BLK kinase while shControl lentivirus codes for unrelated sequences. Top, western blot analysis using antibody to BLK; bottom, western blot analysis using antibody to GAPDH as loading control. (B) The relative BLK mRNA is reduced to half in the silencing line (shBLK)(***P<0,001 based on Student´s test comparing mRNA expression between Daudi cell versus cBLK silencing cells and control silenced cells versus BLK silencing cells). (C) Immunoprecipitates (IPs) of stimulated silenced shBLK cells with anti-IgM, using anti-PLCg2 antibody and interrogated with anti-BANK1 to assay BANK1-PLCg2 association. Quantification of the immunoprecipitates normalized with BANK1 is displayed below the bands. Silencing of BLK leads to less association between BANK1-PLCg2. (D) Kinetics of the association between BANK1-PLCg2 assayed with *in situ* Proximity Ligation (PLA) in control and BLK-silenced cell lines. The association between BANK1-PLCg2 is reduced in silenced cells during resting conditions and after 15 minutes of IgM stimulation (***P≤0,001 based on Student´s test comparing control cells versus BLK silencing cells). The difference of BANK1-PLCg2 association between control and the silenced lines was not significant at 1 minute after stimulation.

We then addressed if elimination of BLK influences the BANK1-PLCg2 interaction. We used commercially available lentiviral particles coding for three BLK-specific siRNAs to silence the kinase in the human B-cell line Daudi. We obtained a substantial reduction of protein and mRNA expression in the silenced cell lines (Figure 5A and B).

Silencing of BLK leads to a reduction of the immunoprecipitation between PLCg2 and BANK1 upon stimulation with IgM (Figure 5C). The kinetics of the interaction determined by proximity ligation follows the previously observed pattern (Figure 3B). The PLA signals reached a maximum at one minute after stimulation and decreased to the basal level after 15 minutes. In non-stimulated cells and after 15 minutes of IgM stimulation the differences in PLA interactions were significant between silencing and control cells (P<0.0001 in *t*-test). At one minute after stimulation the difference did not reach the significance level (P = 0.0561). These results suggest that other kinases are able to compensate for the reduction of BLK during intensive stimulation. They also suggest a role for BLK in the maintenance of a homeostatic modulation of the BANK1-PLCg2 interaction. The depletion of BLK leads to larger fluctuations of the interaction between the two proteins than in the presence of BLK.

### The BANK1-PLCg2 interaction is dependent on the proline rich motif and the phosphorylation of specific tyrosine residues on BANK1

**Figure 6:**
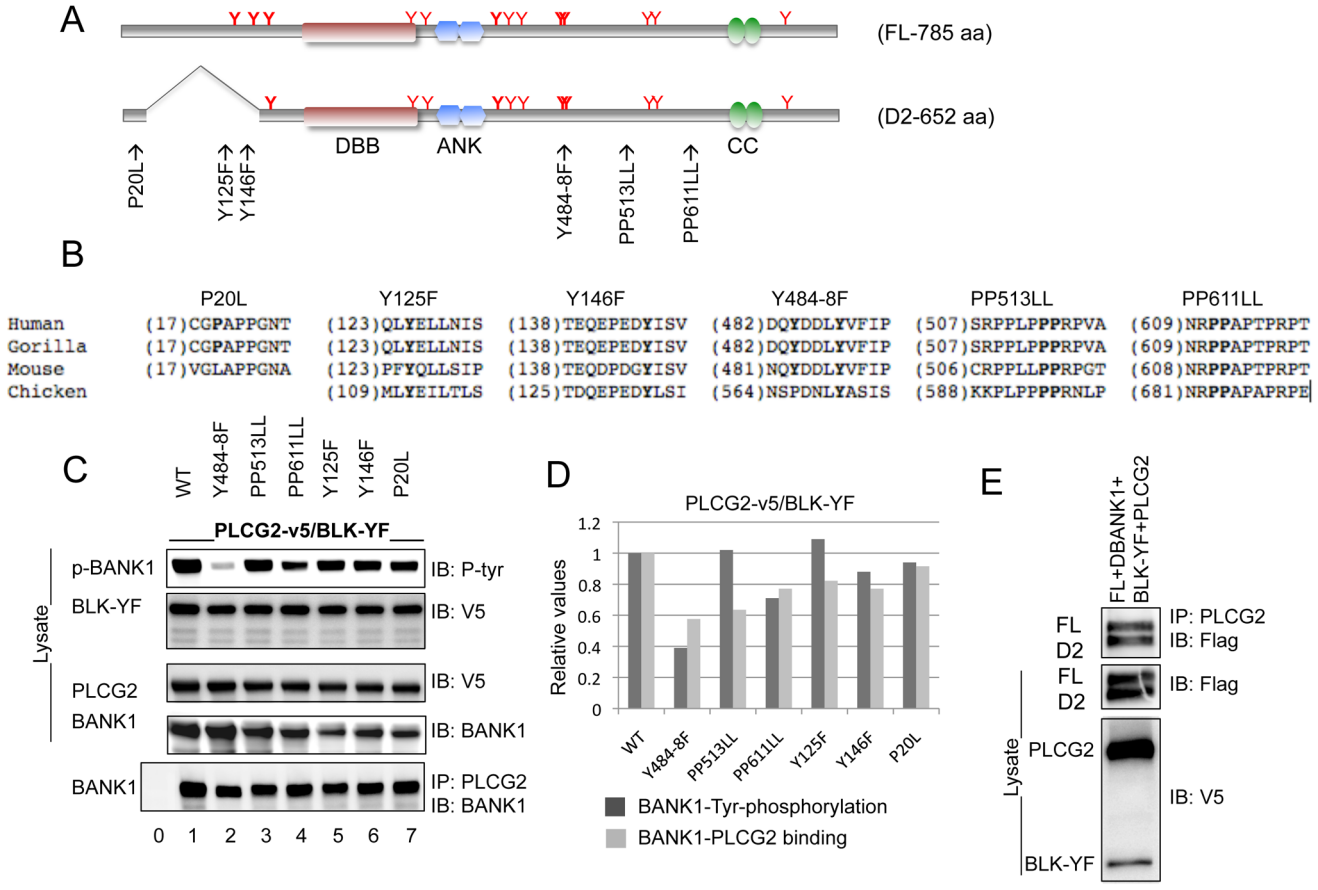
Mutations of specific tyrosine residues and proline rich domains of BANK1 abrogated the association with PLCg2. (A) Schematic representation of the motifs and the structure of the human BANK1 splicing variants and analysis of the motifs affecting the BANK1-PLCg2 binding. The Dof/BCAP/BANK (DBB) motif (amino acids 199–327), the double ankyrin repeat-like (ANK) motifs (amino acids 339–402) and the presumptive coiled coils (CC) region (amino acids 677–705) are indicated. Tyrosine residues susceptible to be phosphorylated are shown by Y. Residues Y125, Y146, Y161, Y416, Y484 and Y488 that predict the putative SH2 binding sites are indicated as bold Y (http://scansite.mit.edu/motifscan_seq.phtml). The positions of the mutated amino acids are indicated above the BANK1 drawing. (B) Alignment of the BANK1 amino acid motifs in different species indicating the mutated residues corresponding to putative SH2 and SH3 binding domains, alignment was done using data downloaded from www.ensembl.org. (C) Phosphorylation and immunoprecipitation analysis of the wild type and mutated forms of BANK1 co-expressed with the constitutively active form of BLK (BLK-YF) and PLCg2. Relative expression of the constructs was monitored by western blot (IB) of an aliquot (1/10) of the transfected HEK293T cell extract. The rest of the lysates were used for immunoprecipitation (IP) using anti-PLCg2 (Abcam) and it is shown in the lower row. Lane 0 represents immunoprecipitation without the IP antibody. (D) Quantification of BANK1 phosphorylation and immunoprecipitation using anti-PLCg2 antibody. Bands of the western blots were quantified using ImageJ program. Values of expression of BANK1 in the lysates (IB:Anti-BANK1) were taken to normalize the results of phosphorylation and immunoprecipitation of BANK1. The relative expression of BANK1 was: 1, 1.03, 0.93, 0.85,0.69, 0,81, and 0.73 from lane 1 to 7 in the lysates interrogated with anti-BANK1. The Y484-8F mutation significantly reduced both the tyrosine phosphorylation of BANK1 and the binding to PLCg2. The PP513LL mutation does not affect the tyrosine phosphorylation of BANK1, however it leads to a decrease in binding to PLCg2. (E) Co-expression in HEK293T cells of the two isoforms of BANK1 renders equivalent recovery of both isoforms in the anti-PLCg2 immunoprecipitate, indicating that exon 2 did not participate in the binding between BANK1 and PLCg2.

BCR stimulation or the co-expression of an active tyrosine kinase induced the phosphorylation of BANK1 and enhanced its association with PLCg2. This suggests that certain tyrosine residues on BANK1 are important for the interaction. The fact that the prey clones retrieved in our Y2H screen coded for phosphotyrosine-binding domains (SH2) reinforced this notion. The full-length isoform of BANK1 (FL) has thirteen potential tyrosine phosphorylation residues. Two of them are absent in the short isoform (D2) lacking the second exon (Figure 6A). We targeted these two residues (Y125 and Y146) and performed a binding assay. In addition, we mutated two adjacent tyrosines (Y484 and Y488) that are predicted to form a strong SH2 binding motif. Because the prey clones also contain proline rich binding motifs (SH3), we analyzed the effect of BANK1 proline substitutions in our binding assay. The mutated sites have a variable degree of conservation on orthologous proteins, thus, the proline P20 is poorly conserved while the sequence surrounding the prolines P611 and P612 is highly conserved (Figure 6B). We expressed the mutated BANK1 proteins in cells transfected with the kinase constitutive active form of BLK (BLK–YF) and PLCg2 (Figure 6C). The association was measured by immunoprecipitation using the anti-PLCg2 antibody and the level of BANK1 tyrosine phosphorylation was estimated with the anti-pan-tyrosine antibody (Figure 6D). Substitution of Y484 and Y488 to F led to an overall decrease of tyrosine phosphorylation of BANK1 (lane 2, first row), the substitution of Y125 did not influence the BLK-mediated phosphorylation and the substitution of Y146 only marginally reduced the level of tyrosine phosphorylation, indicating that Y484 and/or Y488 were phosphorylated by the constitutively active form of BLK while Y125 was not. The BANK1 association to PLCg2 was significantly reduced in the Y484–488 substitution but not completely abolished, which suggested additional binding sites. The PP513LL seemed to represent such an additional binding site because its mutation leads to a reduction of the association. In this case, the association is independent from the overall tyrosine phosphorylation of BANK1 (Figure 6D). We addressed further the interaction of PLCg2 with the natural occurring isoforms of BANK1. Immunoprecipitation of co-expressed BANK1 isoforms (FL and D2) showed as expected that both proteins associated equally to PLCg2 (Figure 6D). Thus, BANK1 has two defined domains, one containing exon 2 that binds to type 2 IP3R [11] and a PLCg2 binding domain composed of a phosphotyrosine motif (Y484–488) that probably binds to the SH2 domains of PLCg2 and a proline rich motif (PP513–514) that probably binds to the SH3 domain of PLCg2. The two domains connect the enzyme responsible for the generation of IP3 (PLCg2) and the receptor of this second messenger (IP3R). The signaling cascade is initiated by BCR-mediated phosphorylation of BANK1.

## Discussion

In the present study, we have demonstrated that the adaptor protein coded by the gene BANK1 interacts physically with a major effector of intracellular signaling, the phosphoinositide-specific phospholipase C gamma 2 (PLCg2). We demonstrate that BANK1-PLCg2 interaction is transient and regulated by engagement of the B-cell receptor (BCR). We show further that the non-receptor kinase BLK modulates this interaction.

The two-hybrid assays show that BANK1 interacts with molecules containing adjacent SH2 and SH3 domains. The clone a-14 is an exception because it lacks these two domains. A reasonable explanation is that these domains have been deleted from a larger clone as a result of recombination events that have taken place at some point in the isolation of the positive clone. These events are relatively common in yeast. It is striking, however, that given that the SH2-SH3 domains are widely represented in interacting proteins only clones of the Src non-receptor kinases and PLCg family members were recovered. It would have been expected that abundant proteins containing SH2 and SH3 motifs such VAV, BTK or CSK would have appeared in the screening. The absence of these proteins indicates that the interaction of BANK1 with the Src kinases and PLCg families of proteins is specific or at least favoured in our assay. Our Y2H screen suggests that the cSH2 and SH3 motifs of PLCg2 are the docking sites for BANK1 (Figure 1). The adaptor molecule B-cell linker (BLNK) also known as SLP65 or BASH, acts downstream of BCR signaling through the assembly of multiple molecules including PLCg2 (for review [33]. This adaptor preferentially interacts with the nSH2 motif of PLCg2 [34], thus, it is likely that each adaptor has specific binding regions to PLCg2.

LYN and BLK are the predominant Src-kinases in human B cells. Their individual contribution to phosphorylation of BANK1 is not known, due to the absence of null mutants. In the chicken B-cell line DT40, LYN is dispensable for the inducible phosphorylation of BANK1 and both molecules associate following a non-canonical SH2 or SH3 interaction [11]. It is likely that due to the redundancy for binding sites and activity of LYN and BLK, the two kinases could alter the correct activity of the PLCg2 complex, either through BANK1 or directly on PLCg2. In fact, both kinases interact biochemically with PLCg2. Using peptides generated in bacteria binding of the murine kinases to PLCg2 was demonstrated [35]. Despite being weak, the interactions only required the non-conserved N-terminal domains of the kinases. We show that lipidation, the modification that ultimately determine the trafficking and sub-cellular location of the kinases, confers specificity to the formation of the complex BANK1-PLCg2. The addition of a *de novo* palmytoylation site or the abrogation of the BLK myristoylated site reduced the interaction. The spatiotemporal coordination of BCR associated multiprotein complexes is still poorly understood but it has been pointed out as an important factor in lymphocyte signal transduction [36].

The cellular response upon BCR stimulation when BLK was not present led to wider fluctuation of the BANK1-PLCg2 interaction, which suggests a function of BLK as a negative homeostatic modulator of the response mediated by the BCR. In mice, a negative role of BLK on BCR signaling has been reported [37]. That report shows that marginal zone B-cells of the BLK knockout mouse are hyperresponsive to BCR stimulation. Recently, we have demonstrated that ectopically expressed BANK1 curbs the trafficking of BLK to the plasma membrane [20]. Thus, conditions that lead to the increase in BANK1 expression could have a double effect in promoting a hyperactivation phenotype if there is also a lack of BLK. One would be a direct effect due to the enhancement of the association between BANK1-PLCg2 and other, the increase of signaling fluctuations by an excess of BANK1 due to the sequestration of the homeostatic BLK to the cytoplasm.

The central finding of the present work is that PLCg2, one of the major molecular switches in B-cell signaling, functionally associates with BANK1 and this interaction is mediated by BLK. The role of PLCg2 in immunity has been extensively studied in model organisms. The knockout mice for PLCg2 show decreased IgM levels and absence of intracellular calcium response to BCR stimulation [16], [38]. Two different gain-of-function mutations of murine PLCg2 lead to severe autoimmunity [18], [39]. B-cells of these mouse mutants, *Ali5 and Ali14, (Ali* for abnormal limbs) showed increased and sustained intracellular calcium flux upon anti-IgM stimulation. For the *Ali5* mutation it has been suggested that the amino acid change D993G removes a negative charge from a critical region of the PLCg2 molecule, leading to reduced repulsion from the inner plasma membrane. As a consequence, this could result in persistent positioning of the mutated protein at the plasma membrane. This spatiotemporal alteration could explain the observed increase in signaling in the *Ali5* mutation. The interaction of BANK1 with PLCg2 could play a similar role in modulating the temporal positioning of the phospholipase to the negatively charged inner plasma membrane. In fact, BANK1 has a remarkable stretch of acidic amino acids just downstream of the binding sites for PLCg2 (aa 561–575) that could influence the positioning of the complex (Figure S6).

BANK1 and BLK have been associated to human autoimmune diseases. In addition, the gene GTPase Ras guanyl releasing protein 3 (RASGRP3) acting in BCR-PLCG2 pathway has been associated to SLE, corroborating the importance of this immune signal transduction pathway in this disease [40]. RASGRP3 is activated by DAG and coupled to the translocation to the plasma membrane [41]. The role of BANK1 in the activation of RASGRP3 has not been established but could be a mechanistic link between BANK1 and BLK and the coupling between the BCR and the PKC and Ras pathways [42], [43].Recently, human dominant inherited deletions affecting the PLCg2 locus linked to cold urticaria and autoimmunity were reported [19]. Although the murine mutations and the human deletion do result in an increased lipase activity, the induction of intracellular calcium signaling results in opposed pathway activation, suggesting that deregulation of PLCg2 can result in complex immunological phenotypes through very different mechanisms.

Together, our results outline a new signaling pathway in B-lymphocytes including the autoimmunity associated genes BANK1, PLCg2 and BLK and they fill a gap in our understanding of the mechanistic relation between associated alleles in human autoimmune diseases and B-cell physiology in particular.

## Materials and Methods

### Y2H screen

Two independent screens were performed as a service by Hybrigenics S.A. (Paris, France). The first one was done using as a bait the human full-length BANK (amino acids 1–785), and the second one using a truncate form of BANK1 (amino acids 331–785) In both screens, the bait constructs were transformed with a Human leukocyte and mononuclear cell library (Hybrigenics). A total of 6×10^6^ interactions were tested in each screen.

### Cloning, expression vectors and mutagenesis

Human BANK1 cDNA was PCR-amplified from human peripheral blood mononuclear cells and cloned into the expression vectors pcDNA3.1D/V5-His-TOPO (Invitrogen, Boston, MA, USA) and pires2-EGFP (Clontech, Palo Alto, CA). The coding sequences of BLK, ATG4b and CD163 were amplified from BJAB cells' cDNA and cloned into pcDNA3.1D/V5-His-TOPO (Invitrogen). Fluorescent fusion proteins were added in frame at the C-terminal using the cloning sites NotI/XbaI. BANK1 and BLK mutants were generated by site-directed mutagenesis. The constitutively active form of BLK (BLK-YF) has a substitution of a tyrosine residue to phenylalanine in the C-terminal regulatory domain (Y501F), the kinase dead form (BLK-KL) was generated by the K269L substitution in the catalytic site. PLCG2 and LYN were amplified from the I.M.A.G.E. full length cDNA clone IRAUp969G0437D and pME-LYN [11], respectively, and cloned into pcDNA3.1D/V5-His-TOPO (Invitrogen). (For detailed information, see methods S1 & S2 online). All clones were confirmed by sequencing.

### Cell culture and transfections

Daudi and embryonic kidney HEK293T cells were each maintained in RPMI 1640 medium and Dulbecco's modified Eagle's medium containing Glutamax (Invitrogen) supplemented with 10% fetal bovine serum (Invitrogen). HEK293T cells (3*10^6^) were transiently transfected with 20 uL Lipofectamine 2000 (Invitrogen) and 8 ug of each DNA vector following the manufacturer instructions. The analysis of cells was performed 48 hours after transfections.

### Antibodies

The antibodies used for immunoprecipitation and western blot were: Mouse anti-V5 (Invitrogen), mouse anti-Phospho-Tyrosine #9411 (Cell signaling, Beverly, MA), mouse anti-PLCG2 ab89625 (Abcam, Cambridge, UK), rabbit anti-BANK1-ET52, rabbit anti-BANK1 HPA037002 (Sigma, St. Louis, Mo, USA), mouse anti-BLK H00000640-M02 (Abnova, Heidelberg, Germany) and chicken anti-GAPDH SAB3500247 (Sigma), anti-rabbit and anti-mouse-HRP (Zymed,, San Francisco, CA, USA) anti-Chicken IgY-HRP (Sigma).

### Co-immunoprecipitation and Western blot analysis

For immunoprecipitation, HEK293T transfected cells (10*10^6^) or Daudi cells (3*10^7^) were solubilized in NP-40 lysis buffer containing 1% NP-40, 50 mM Tris pH 7.4, 150 mM NaCl, 2 mM Na3VO4, 1 mM PMSF and protease and phosphatase inhibitor cocktails (Roche) for 10 minutes on ice and centrifuged at 20000 g for 10 minutes at 4°C. An aliquot of each lysate was saved for input analysis and the remaining lysates were immunoprecipitated with 3 ug of anti-PLCG2 (Abcam) previously bound to 50 uL protein G Dynabeads (Invitrogen) for 3 hours at 4°C with rotation. Dynabeads-Ab-Ag complexes were washed 3 times with ice cold Dulbecco's phosphate-buffered saline (DPBS) including proteases and phosphatase inhibitors and eluted in 30 uL elution buffer containing NuPAGE LDS Sample Buffer 1× and NuPAGE reducing agent 1× (Invitrogen) by heating at 70°C for 10 minutes. Lysates and immunoprecipitates were separated by 4–15% gradient SDS-PAGE gels (BioRad, Barcelona, Spain), transferred to PVDF membranes (Biorad) and detected with the appropriate antibodies on a ECL system.

### Cell stimulation and silencing

For stimulation, Daudi cells were washed with DPBS and changed to RPMI1640 medium without FBS two hours before addition of the stimulus. Cells were resuspended and stimulated in Opti-MEM I medium (Invitrogen) with 10 ug/mL goat F(ab')2 anti-human IgM (Southernbiotech, Birmingham, Alabama, USA) at 37°C for the indicated times. The cells were transferred to ice to stop the stimulation. Before co-immunoprecipitation, the cells were washed with ice-cold DPBS and lysed with NP-40 lysis buffer. For silencing, Daudi cells were transduced with Blk shRNA Lentiviral Particles (cat no. sc-39227-V) or control scrambled shRNA Lentiviral Particles (cat no. sc-108080, Santa Cruz Biotechnology Santa Cruz, CA, USA) following the manufacturer instructions.

### Microscopy

Cells were grown and transfected on Lab-Tek chamber slides coated with poly-D-lysine (Beckton Dickinson, Oxford, UK). Twenty-four hours after transfection cells were fixed at room temperature for 20 minutes with 3.7% paraformaldehyde in a buffer containing PBS with 0.18% Triton-X. Fluorescent fusion proteins were visualized directly after fixation, FX enhancer treatment (Invitrogen) and mounted with Vectashield (Vector Lab. Peterborough, UK) or SlowFade Gold Antifade Reagent (Invitrogen) containing DAPI. Confocal microscopy was performed using a Zeiss 510 Meta confocal scanning microscope with a Zeiss plan-Apochromat 63× oil-immersion objective (Zeiss, Stockholm, Sweden). Dual- or triple-color images were acquired by consecutive scanning with only 1 laser line active per scan to avoid cross-excitation. Image analysis was carried out using ImageJ software.

### *In situ* proximity ligation assay (PLA)

Daudi cells were seeded into an 8-well culture slide coated with polylysine. Cells were grown for 4 hours and stimulated with 10 ug/mL goat F(ab')2 anti-human IgM (Southernbiotech) diluted in Opti-MEM I medium at 37°C for the indicated times. Stimulation was stopped by fixation of cells with paraformaldehyde solution at 4% final concentration. Slides were incubated for 20 minutes at RT, washed with PBS-Tween 0.05% and permeabilized with methanol:acetone (1∶1) for 10 minutes at −20°C. After permeabilization, cells were washed twice with PBS-Tween 0.05% and residual liquid dried at RT.

Proximity ligation assay was done with the Duolink II kit according to the manufactureŕs protocol (Olink Bioscience, Uppsala, Sweden)). Briefly, slides were incubated with blocking solution in a pre-heated humidity chamber for 30 minutes at 37°C. Cells were incubated with rabbit anti-human BANK1 ET52 [20], alternatively rabbit anti-human BANK1 HPA037002 (Sigma) together with mouse anti-human PLCG2 antibody (ab89625, Abcam) overnight in a humidity chamber at 4°C. After incubation, slides were washed twice and incubated with mouse minus and rabbit plus PLA probes for 1 h at 37°C. Ligation was carried out for 30 minutes at 37°C and amplification for 100 min at 37°C. Finally, slides were washed, dried at RT in the dark and mounted with SlowFade Gold Antifade Reagent (Invitrogen) containing DAPI for nuclei staining.

The images were taken with a confocal microscope and the quantification was done using the free software BlobFinder (Centre for Image Analysis, Uppsala University, Uppsala, Sweden) (for PC) or alternatively using our own developed plug-in for ImageJ (to be used on Mac computers, see methods S1 & S2 and Figure S3).

## Supporting Information

Figure S1 **Domain mapping autoactivator assay for BANK1.** A) Solid grown assay on DO-3 medium of transformants carrying coding fragments of BANK1. B) Summary of the results of the assay.Six fragments were amplified by PCR using long-primers, which contain an homologous region of 50 nt with the DNA-binding domain plasmid (DBD)pB27 and 20 nt from the bait fragment. The fragments were subsequently transformed together with linearized pB27 bait vector into yeast cells for cloning by gap-repair. The empty activation domain (AD) plasmid pP7 was co-transformed. Transformants with a positive homologous recombination event between bait plasmid and PCR fragment were selected on solid DO-2 medium (-Trp, -Leu, selective for the presence of both DBD and AD fusions).The interaction assay uses the His3 reporter gene that allows the yeast to grow on a medium lacking histidine. Autoactivation of the bait fragments is assayed in presence of 3-Aminotriazole (3-AT), a competitive inhibitor of the product of His3 (Vojtek et al.1993).Eight transformants from each fragment were tested for their autoactivation in a solid growth assay (robot calibrated drops) on DO-3 medium (-Trp, -Leu, -His) supplemented with increasing concentrations of 3-AT (0, 1, 5, 10, 50 mM). The original bait fragment (hgx2518v1_pB27) was tested as positive control. Yeast cells transformed with empty ADvector and empty DBD vector as well as empty AD vector and open DBD vector, respectively, were tested as negative controls.The full-length protein (hgx2518v1_pB27 and fragment 1) as well as the fragment 3 (aa 1–330) are strongly autoactivating the Y2H system. Fragments 2 (aa 1–555) and 4 (aa 170–785) significantly autoactivate the Y2H system and fragments 5 (aa 331–785) and 6 (aa 579–785) do not autoactivate the Y2H system even on medium with the lowest selection pressure. The fragment 5 was used for a second Y2H screening.**References** Vojtek et al., Cell, 1993, 74(1):205–14
(TIF)

Figure S2 **Ectopically expressed BANK1 and ATG4b proteins in human HEK293T cells show co-localization in punctate structures (arrows).** The coding sequences were fused to green fluorescence protein (gfp) or mCherry (che) by the carboxy- termini.
(TIF)

Figure S3 **Validation of methodologies to quantify cells and PLA signals from microscopy images.** (A) Correlation between nuclei number counted in 36 slices by BlobFinder versus our plug-in for ImageJ. (B) Correlation between the PLA signal counted by BlobFinder and the plug-in developed to be used with ImageJ.
(DOCX)

Figure S4 **(A)**. In situ PLA of HEK293T cells co-transfected with combinations of the construct pPLCg2, pBANK1 and GFP. a) Cells transfected with pPLCg2-V5, pBANK1 and pGFP. PLA signals were detected using anti-BANK1-ET52 and anti-PLCg2. b) Cells were transfected with the same plasmid mix as in a) and the PLA reaction was developed using anti-BANK1 (Sigma-HPA) and anti-PLCg2. c) Transfection omitting pBANK1 and d) Transfection omitting pPLCg2. PLA, in c) and d) done with anti-BANK1 (Sigma-HPA) and anti-PLCg2. Cells were grown on Lab-Tek chamber slides. A total plasmid amount of 1.2 ug per chamber was transfected using Lipofectamine 2000 (Invitrogen). The plasmid expressing GFP was used to compensate the total amount of DNA in control experiments and to estimate the transfection efficiency. Twenty four hours after transfection cells were fixed at room temperature for 20 min with 4% paraformaldehyde in PBS/0.18% Triton-X and permeabilized on ice-cold 50∶50 methanol-acetone at −20°C for 10 min. The PLA reactions were performed following the DUOLINK II protocol with the anti-rabbit plus and anti-mouse minus PLA probes and the signal detected with **Duolink II Detection Reagents Orange.** The preparations were counterstained with DAPI and mounted on microscope slides using Vectashield (Vector Laboratories). Images were acquired with a Zeiss Axiovert 200 M epifluorescence microscope (Carl Zeiss).**(B)** Confocal images of an EBV-transformed human lymphoblastoid B cell line showing molecular proximity between endogenous BANK1 and PLCg2 proteins. The staining was done using *in situ* PLA with rabbit anti-BANK1 (Sigma) and mouse anti PLCg2 (Abcam). Nuclei are stained with DAPI in blue. Upper panel, non-stimulated cells. Low panel, cells stimulated for 20 minutes with the specific anti-human IgM F(ab)2 antibody (Southern Biotech).
(TIF)

Figure S5 **Co-immunoprecipitation in Daudi B-cells of BANK1 and PLCG2 using the antibody against BANK1 followed by interrogation with anti-PLCg2.** The complex is formed after IgM stimulation and it is absent in extracts from non stimulated cells (NS). The gel contains extracts from transfected HEK293 cells with constructs coding for Flag-BANK1 and PLCg2-V5 to accurately determine the mobility of the endogenous proteins.
(TIF)

Figure S6 **Hydrophobic cluster analysis of BANK1 performed at** http://bioserv.impmc.jussieu.fr/.The mutated tyrosines Y484 and Y488 are indicated by arrows. The mutated prolines P513 and P514 are indicated by a circle. The acidic negative charge cluster is indicated by a bracket.Symbols are used for amino acids with peculiar properties (star  =  proline, black diamond  =  glycine, open square  =  threonine, dotted square  =  serine).
(TIF)

Table S1 **Complete set of prey proteins obtained in the Y2H screen using the truncated form of BANK1 (aa 331–785).**
(XLSX)

Method S1 (**A**). Primers used for cloning and fluorescence tagging of the expression constructsNote: Bases modified for cloning are indicated in uppercases and the start codons in italics.(**B**) Primers used for directed mutagenesis.Mutated nucleotides are showed in lowercases.
(DOC)

Method S2 **Confocal images were taken with a 40× objective and a pinhole of 3 in a LS 510 Zeiss microscope.**Quantification of cells by DAPI staining:run("Enhance Contrast", "saturated = 50");run("8-bit");run("8-bit");run("Make Binary");run("Erode");run("Ultimate Points");run("Make Binary");run("Analyze Particles...", "size = 0-Infinity circularity = 0.00–1.00 show = Nothing display clear include summarize record add");Quantification of PLA signals:run("Sharpen");run("Gamma...", "value = 2.500");run("Make Binary");run("Analyze Particles...", "size = 0.10–3.00 circularity = 0.00–1.00 show = Nothing display clear include summarize record add");
(DOCX)
