# Supplementary figures and images for: BANK1 and BLK Act through Phospholipase C Gamma 2 in B-Cell Signaling
Source: PLoS One. 2013 Mar 26;8(3):e59842. doi: 10.1371/journal.pone.0059842 (PMC3608554; doi:10.1371/journal.pone.0059842)

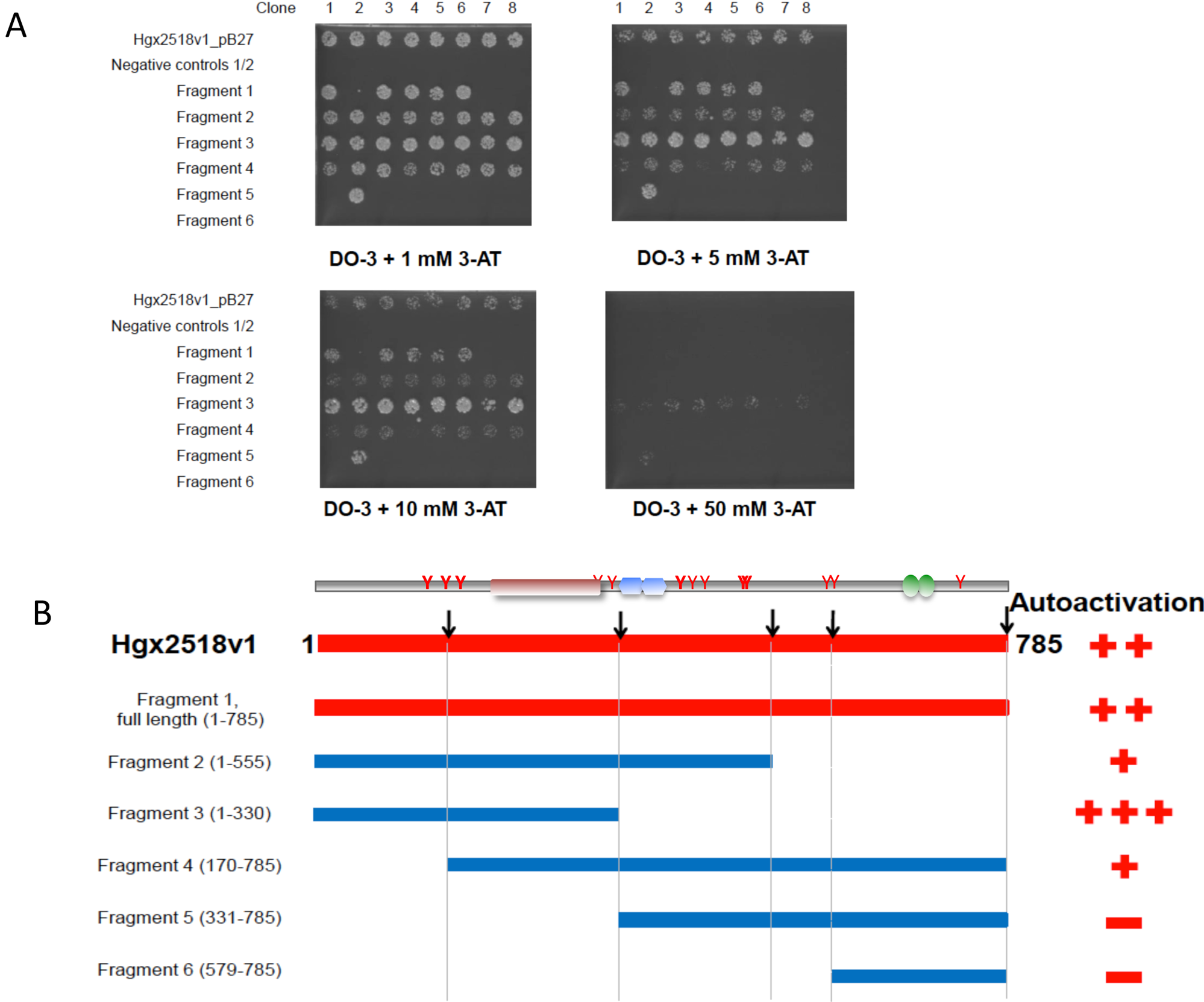

Supplement: Figure S1 — Domain mapping autoactivator assay for BANK1. A) Solid grown assay on DO-3 medium of transformants carrying coding fragments of BANK1. B) Summary of the results of the assay.Six fragments were amplified by PCR using long-primers, which contain an homologous region of 50 nt with the DNA-binding domain plasmid (DBD)pB27 and 20 nt from the bait fragment. The fragments were subsequently transformed together with linearized pB27 bait vector into yeast cells for cloning by gap-repair. The empty activation domain (AD) plasmid pP7 was co-transformed. Transformants with a positive homologous recombination event between bait plasmid and PCR fragment were selected on solid DO-2 medium (-Trp, -Leu, selective for the presence of both DBD and AD fusions).The interaction assay uses the His3 reporter gene that allows the yeast to grow on a medium lacking histidine. Autoactivation of the bait fragments is assayed in presence of 3-Aminotriazole (3-AT), a competitive inhibitor of the product of His3 (Vojtek et al.1993).Eight transformants from each fragment were tested for their autoactivation in a solid growth assay (robot calibrated drops) on DO-3 medium (-Trp, -Leu, -His) supplemented with increasing concentrations of 3-AT (0, 1, 5, 10, 50 mM). The original bait fragment (hgx2518v1_pB27) was tested as positive control. Yeast cells transformed with empty ADvector and empty DBD vector as well as empty AD vector and open DBD vector, respectively, were tested as negative controls.The full-length protein (hgx2518v1_pB27 and fragment 1) as well as the fragment 3 (aa 1–330) are strongly autoactivating the Y2H system. Fragments 2 (aa 1–555) and 4 (aa 170–785) significantly autoactivate the Y2H system and fragments 5 (aa 331–785) and 6 (aa 579–785) do not autoactivate the Y2H system even on medium with the lowest selection pressure. The fragment 5 was used for a second Y2H screening.References Vojtek et al., Cell, 1993, 74(1):205–14 (TIF) [file pone.0059842.s001.tif]

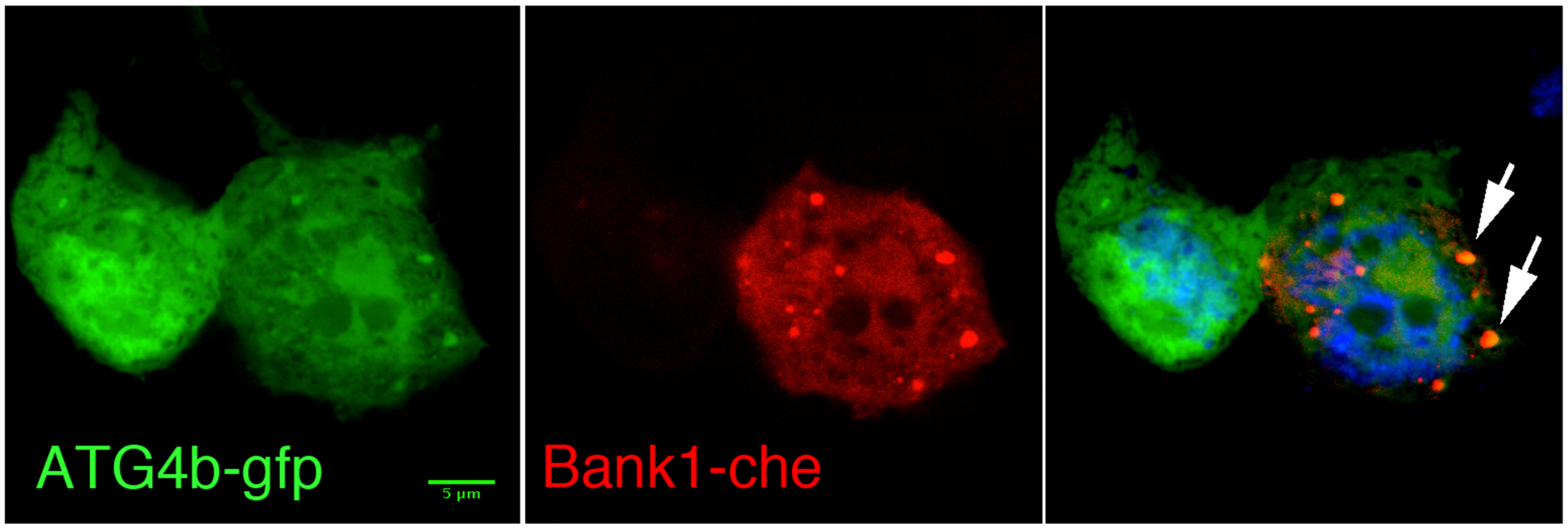

Supplement: Figure S2 — Ectopically expressed BANK1 and ATG4b proteins in human HEK293T cells show co-localization in punctate structures (arrows). The coding sequences were fused to green fluorescence protein (gfp) or mCherry (che) by the carboxy- termini. (TIF) [file pone.0059842.s002.tif]

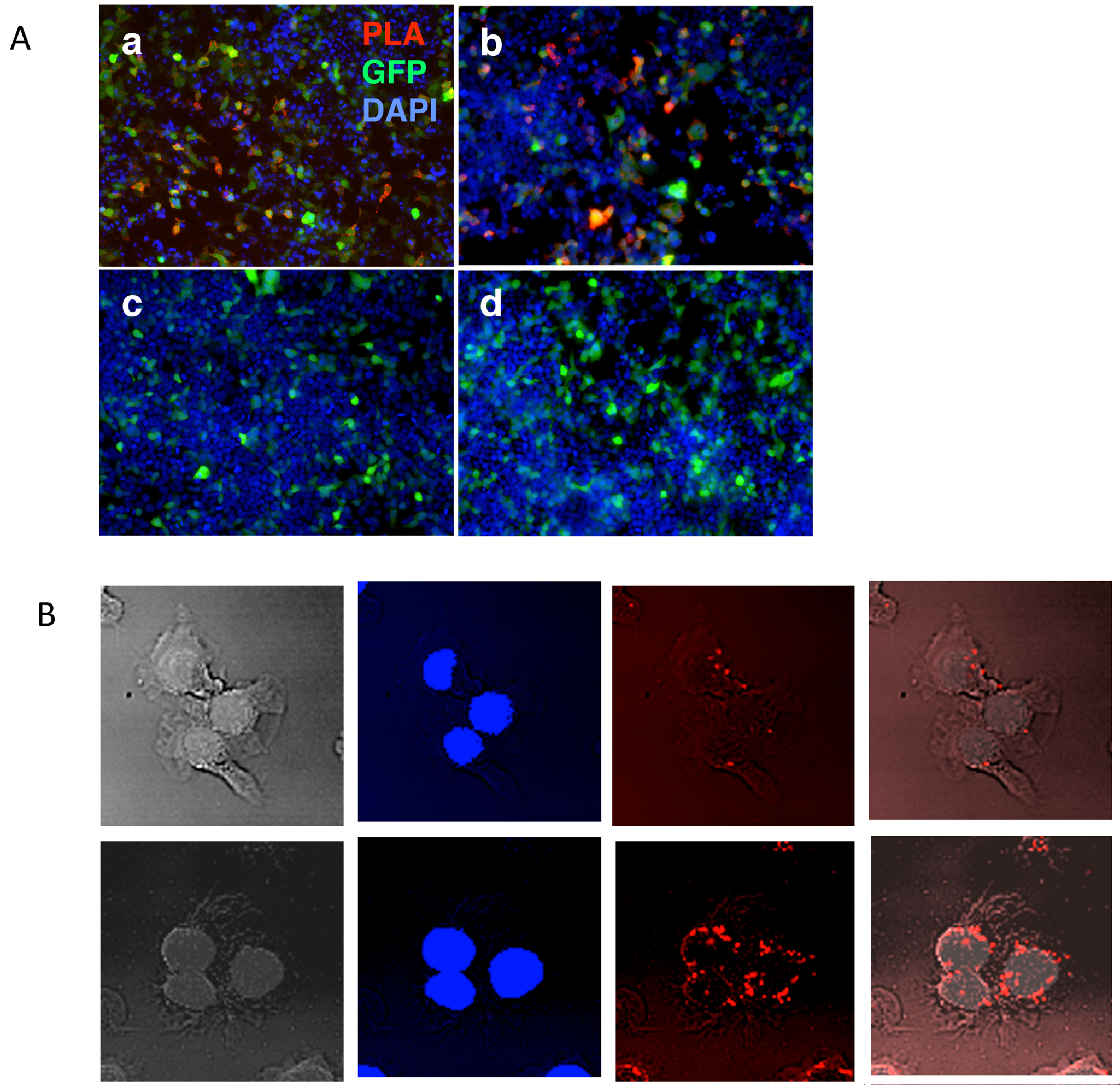

Supplement: Figure S4 — (A). In situ PLA of HEK293T cells co-transfected with combinations of the construct pPLCg2, pBANK1 and GFP. a) Cells transfected with pPLCg2-V5, pBANK1 and pGFP. PLA signals were detected using anti-BANK1-ET52 and anti-PLCg2. b) Cells were transfected with the same plasmid mix as in a) and the PLA reaction was developed using anti-BANK1 (Sigma-HPA) and anti-PLCg2. c) Transfection omitting pBANK1 and d) Transfection omitting pPLCg2. PLA, in c) and d) done with anti-BANK1 (Sigma-HPA) and anti-PLCg2. Cells were grown on Lab-Tek chamber slides. A total plasmid amount of 1.2 ug per chamber was transfected using Lipofectamine 2000 (Invitrogen). The plasmid expressing GFP was used to compensate the total amount of DNA in control experiments and to estimate the transfection efficiency. Twenty four hours after transfection cells were fixed at room temperature for 20 min with 4% paraformaldehyde in PBS/0.18% Triton-X and permeabilized on ice-cold 50∶50 methanol-acetone at −20°C for 10 min. The PLA reactions were performed following the DUOLINK II protocol with the anti-rabbit plus and anti-mouse minus PLA probes and the signal detected with Duolink II Detection Reagents Orange. The preparations were counterstained with DAPI and mounted on microscope slides using Vectashield (Vector Laboratories). Images were acquired with a Zeiss Axiovert 200 M epifluorescence microscope (Carl Zeiss).(B) Confocal images of an EBV-transformed human lymphoblastoid B cell line showing molecular proximity between endogenous BANK1 and PLCg2 proteins. The staining was done using in situ PLA with rabbit anti-BANK1 (Sigma) and mouse anti PLCg2 (Abcam). Nuclei are stained with DAPI in blue. Upper panel, non-stimulated cells. Low panel, cells stimulated for 20 minutes with the specific anti-human IgM F(ab)2 antibody (Southern Biotech). (TIF) [file pone.0059842.s004.tif]

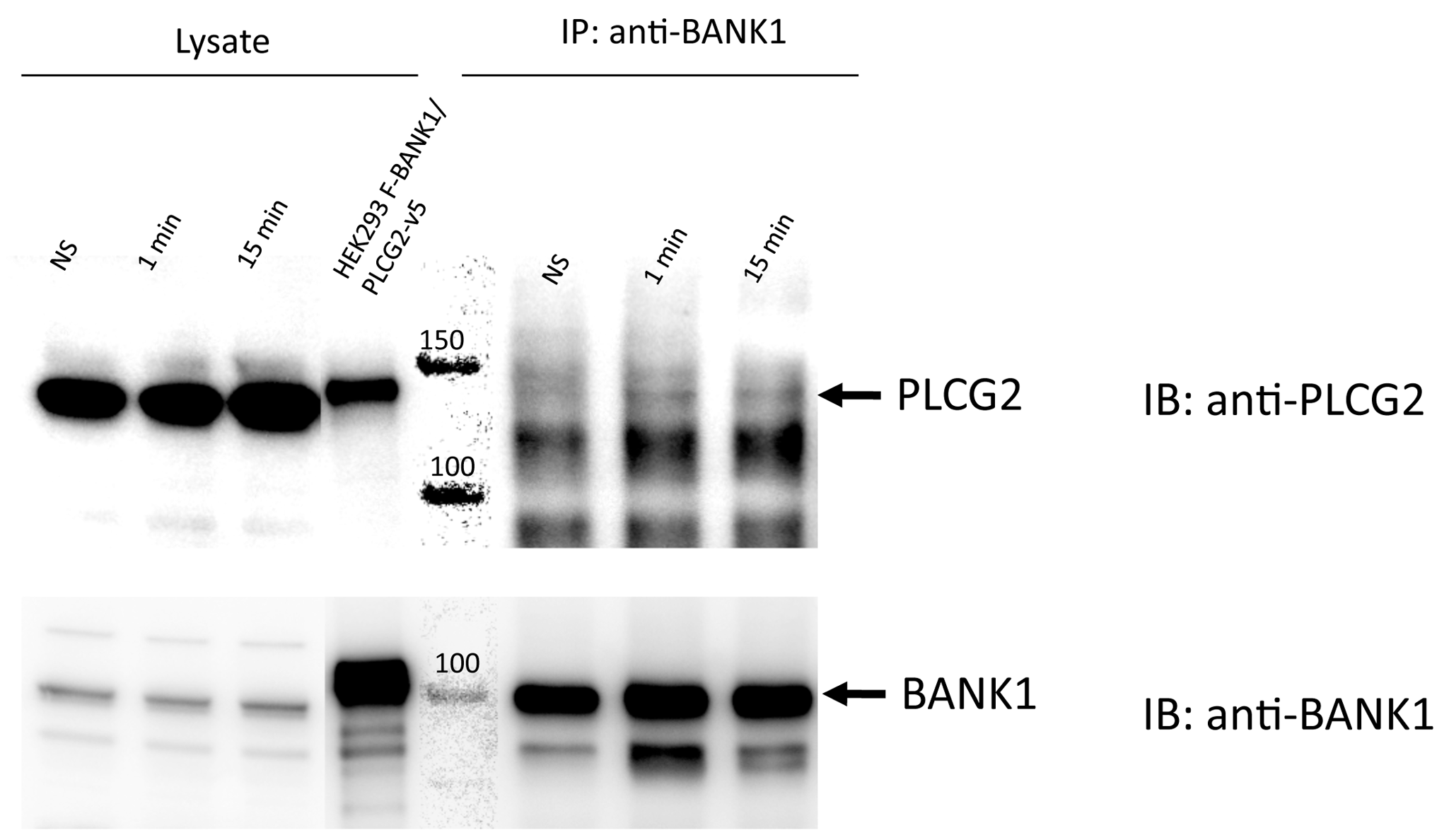

Supplement: Figure S5 — Co-immunoprecipitation in Daudi B-cells of BANK1 and PLCG2 using the antibody against BANK1 followed by interrogation with anti-PLCg2. The complex is formed after IgM stimulation and it is absent in extracts from non stimulated cells (NS). The gel contains extracts from transfected HEK293 cells with constructs coding for Flag-BANK1 and PLCg2-V5 to accurately determine the mobility of the endogenous proteins. (TIF) [file pone.0059842.s005.tif]

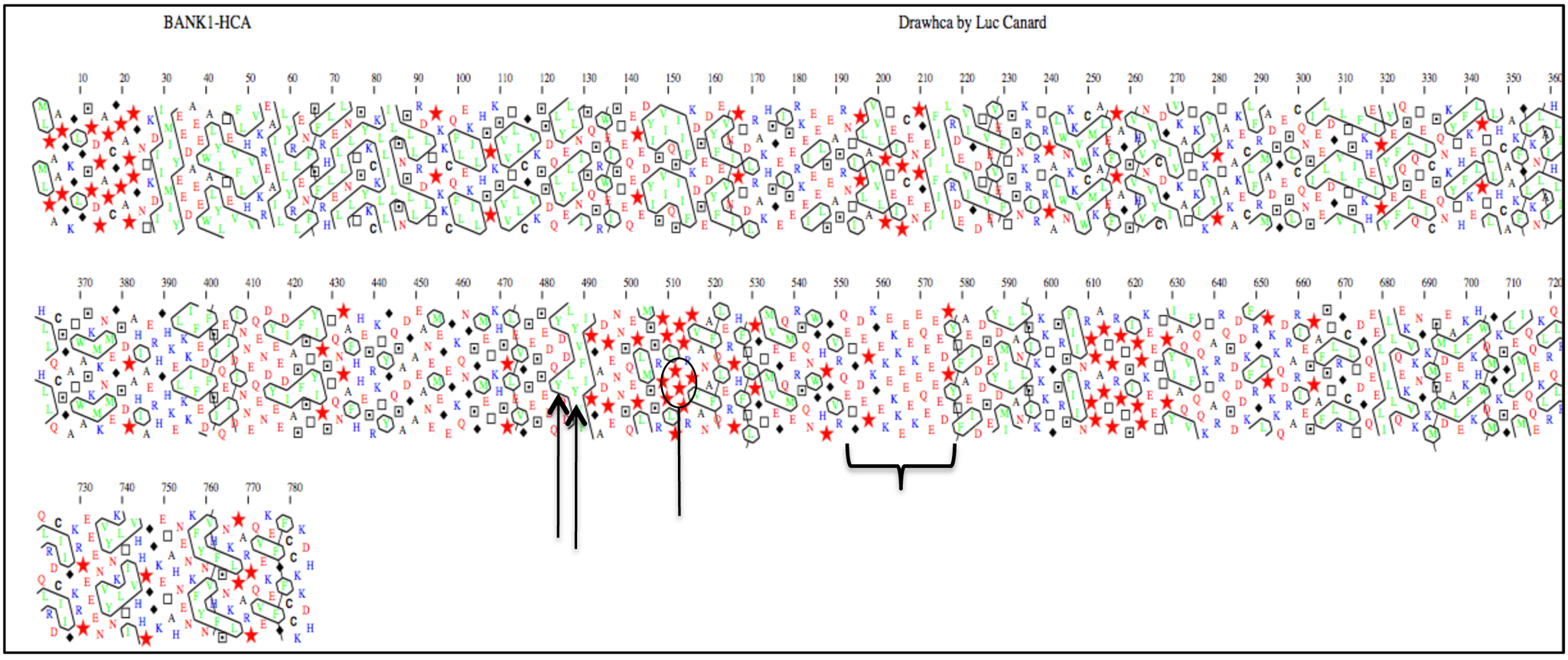

Supplement: Figure S6 — Hydrophobic cluster analysis of BANK1 performed at http://bioserv.impmc.jussieu.fr/.The mutated tyrosines Y484 and Y488 are indicated by arrows. The mutated prolines P513 and P514 are indicated by a circle. The acidic negative charge cluster is indicated by a bracket.Symbols are used for amino acids with peculiar properties (star = proline, black diamond = glycine, open square = threonine, dotted square = serine). (TIF) [file pone.0059842.s006.tif]
