# Supplementary material for: BANK1 and BLK Act through Phospholipase C Gamma 2 in B-Cell Signaling
Source: PLoS One. 2013 Mar 26;8(3):e59842. doi: 10.1371/journal.pone.0059842 (PMC3608554; doi:10.1371/journal.pone.0059842)

**Supplementary Figure S3.** Validation of methodologies to quantify cells and PLA signals from microscopy images.

(A) Correlation between nuclei number counted in 36 slices by BlobFinder versus our plug-in for ImageJ.

| **Correlation nuclei** |  |
| --- | --- |
| Number of XY Pairs | 36 |
| Pearson r | 0,9935 |
| 95% confidence interval | 0.9872 to 0.9967 |
| P value (two-tailed) | P<0.0001 |
| P value summary | *** |
| Is the correlation significant? (alpha=0.05) | Yes |
| R squared | 0,9871 |

(B) Correlation between the PLA signal counted by BlobFinder and the plug-in developed to be used with ImageJ.

| **Correlation PLA** |  |
| --- | --- |
| Number of XY Pairs | 36 |
| Pearson r | 0,9360 |
| 95% confidence interval | 0.8772 to 0.9671 |
| P value (two-tailed) | P<0.0001 |
| P value summary | *** |
| Is the correlation significant? (alpha=0.05) | Yes |
| R squared | 0,8761 |


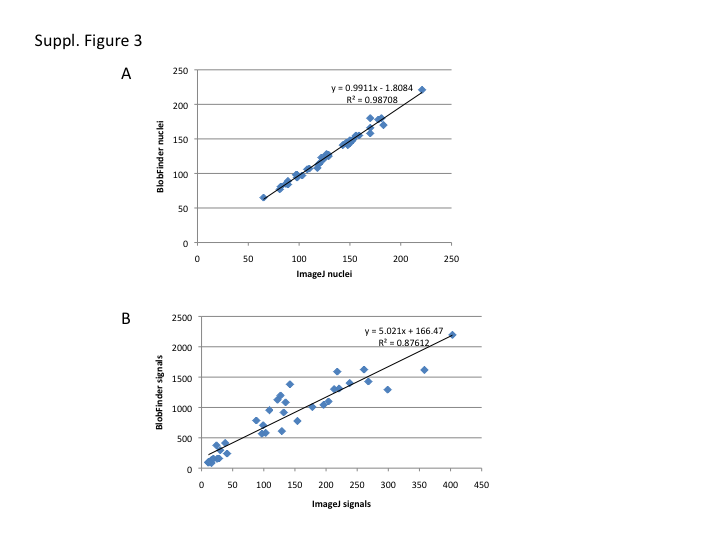

Supplement: Figure S3 — Validation of methodologies to quantify cells and PLA signals from microscopy images. (A) Correlation between nuclei number counted in 36 slices by BlobFinder versus our plug-in for ImageJ. (B) Correlation between the PLA signal counted by BlobFinder and the plug-in developed to be used with ImageJ. (DOCX) [file pone.0059842.s003.docx]
