# Supplementary material for: BANK1 and BLK Act through Phospholipase C Gamma 2 in B-Cell Signaling
Source: PLoS One. 2013 Mar 26;8(3):e59842. doi: 10.1371/journal.pone.0059842 (PMC3608554; doi:10.1371/journal.pone.0059842)
Supplement: Method S1 — (A). Primers used for cloning and fluorescence tagging of the expression constructsNote: Bases modified for cloning are indicated in uppercases and the start codons in italics.(B) Primers used for directed mutagenesis.Mutated nucleotides are showed in lowercases. (DOC) [file pone.0059842.s008.doc]

**Supplementary methods 1a**. Primers used for cloning and fluorescence tagging of the expression constructs

| Constructs | Name | Sequence |
| --- | --- | --- |
| pcDNA-BLK-v5 | f-BLK | 5´-CACC*atg*gggctggtaagtagc-3´ |
|  | r-BLK | 5´-gggctgcagctcgtactgcc-3´ |
|  |  |  |
| pcDNA-BANK | f-BANK | 5´-CACCtcaaccgccaca*atg*ctgccagca-3´ |
|  | r-BANK | 5´-ataataaccttctttaatgatctttcttgc-3 |
|  |  |  |
| pIRES-Flag-BANK | f-FLAG-k | 5´-cacaaccatggattacaaggatgacgacg-3´ |
|  | f-FLAG-m | 5´-attacaaggatgacgacgataagatgctgc-3´ |
|  | f-FLAG-BANK | 5´-cgacgataag*atg*ctgccagcagcgccag-3´ |
|  | r-BANK-H1 | 5´-AGGATccttctttaatgatctttc-3´ |
|  |  |  |
| pcDNA-Plcg2 | PLC-f | 5´-CACC*atg*tccaccacggtcaatg-3´ |
|  | PLC-r | 5´-tgagtaaaacttgctgttgc-3´ |
|  |  |  |
| pcDNA-CD163 | CD163f | 5´-CACC*atg*agcaaactcagaatg-3´ |
|  | CD163r | 5´-taaattcccattttccttttcagt-3´ |
|  |  |  |
| pcDNA-ATG4b | ATGbf | 5´-CACC*atg*gacgcagctactctgacc-3´ |
|  | ATGbr | 5´-aagggacaggatttcaaagtc-3´ |
|  |  |  |
| pcDNA-Lyn | YAMF | 5´-CACC*atg*ggatgtataaaatca-3´ |
|  | YAMR | 5´-aggctgctgctggtattgccct-3´ |
|  |  |  |
| pcDNA-FP-tag | Not-EYFP | 5´-ggGCGGCCGC*atg*gtgagcaagggcga-3´ |
|  | Xba-EYFP | 5´-ggTCTAGActcttgtacagctcgtccat-3´ |

Note: Bases modified for cloning are indicated in uppercases and the start codons in italics.

**Supplementary methods 1b** Primers used for directed mutagenesis

| Constructs | Name | Sequence |
| --- | --- | --- |
| pcDNA-BLK-KL | BLKk275Lf | 5´-AGGTGGCCATTctGACGCTGAAGGAGGGA-3´ |
|  | BLKk275Lr | 5´-CTTCAGCGTCagAATGGCCACCTTCATG-3´ |
|  |  |  |
| pcDNA-BLK-YF | BLKY501Ff | 5´-CGGCCACCGAGCGGCAGTtCGAGCTGCAGC-3´ |
|  | BLKY501Fr | 5´-GGGCTGCAGCTCGaACTGCCGCTCGGTGGC-3´ |
|  |  |  |
| BANK-P20L | BankP20Lf | 5´-CTGCGGCCtAGCGCCCCCAGGAAATAC-3´ |
|  | BankP20Lr | 5´-TCCTGGGGGCGCTaGGCCGCAGGGG-3´ |
|  |  |  |
| BANK-Y125F | BankY125f | 5´-TCAGCTCTtTGAATTACTAAATATC-3´ |
|  | BankY125r | 5´-GTAATTCAaAGAGCTGATCTGAACTC-3´ |
|  |  |  |
| BANK-Y146F | BANKY146f | 5´-TGAAGACTtCATCTCTGTAATCCAG-3´ |
|  | BANKY146r | 5´-TACAGAGATGaAGTCTTCAGGTTCCT-3´ |
|  |  |  |
| BANK-Y484-8F | BANKYYf | 5´-CCAGTtTGATGACTTGTtTGTGTTCATTCCT-3´ |
|  | BANKYYr | 5´-TGAACACAaACAAGTCATCAaACTGGTC-3´ |
|  |  |  |
| BANK-PP513LL | Bpp513f | 5´-TGACCTCCTCTCCtCCtGCCGCGACCT-3´ |
|  | Bpp513r | 5´-GCTACAGGTCGCGGCaGGaGGAGAGGAGG-3´ |
|  |  |  |
| BANK-PP611LL | Bpp611r | 5´-ATAAATAGACtTCtTGCCCCCACACCCCGA-3´ |
|  | Bpp611f | 5´-GTGTGGGGGCAaGAaGTCTATTTATAATG-3´ |

Mutated nucleotides are showed in lowercases.
