# Supplementary material for: BANK1 and BLK Act through Phospholipase C Gamma 2 in B-Cell Signaling
Source: PLoS One. 2013 Mar 26;8(3):e59842. doi: 10.1371/journal.pone.0059842 (PMC3608554; doi:10.1371/journal.pone.0059842)
Supplement: Method S2 — Confocal images were taken with a 40× objective and a pinhole of 3 in a LS 510 Zeiss microscope.Quantification of cells by DAPI staining:run("Enhance Contrast", "saturated = 50");run("8-bit");run("8-bit");run("Make Binary");run("Erode");run("Ultimate Points");run("Make Binary");run("Analyze Particles...", "size = 0-Infinity circularity = 0.00–1.00 show = Nothing display clear include summarize record add");Quantification of PLA signals:run("Sharpen");run("Gamma...", "value = 2.500");run("Make Binary");run("Analyze Particles...", "size = 0.10–3.00 circularity = 0.00–1.00 show = Nothing display clear include summarize record add"); (DOCX) [file pone.0059842.s009.docx]

**Supplementary method 2.**

Confocal images were taken with a 40X objective and a pinhole of 3 in a LS 510 Zeiss microscope.

Quantification of cells by DAPI staining:

run("Enhance Contrast", "saturated=50");

run("8-bit");

run("8-bit");

run("Make Binary");

run("Erode");

run("Ultimate Points");

run("Make Binary");

run("Analyze Particles...", "size=0-Infinity circularity=0.00-1.00 show=Nothing display clear include summarize record add");

Quantification of PLA signals:

run("Sharpen");

run("Gamma...", "value=2.500");

run("Make Binary");

run("Analyze Particles...", "size=0.10-3.00 circularity=0.00-1.00 show=Nothing display clear include summarize record add");
